# Dust Monitors in JET with ITER-like Wall for Diagnosis of Mobilized Particles and Co-Deposited Layers

**DOI:** 10.3390/ma15238353

**Published:** 2022-11-24

**Authors:** Stjepko Fazinić, Georgios Provatas, Iva Božičević Mihalić, Tonči Tadić, Marek Rubel, Justyna Grzonka, Per Petersson, Anna Widdowson, Sunwoo Moon, Elzbieta Fortuna-Zaleśna

**Affiliations:** 1 Division of Experimental Physics, Ruđer Bošković Institute, Bijenička c. 54, 10000 Zagreb, Croatia; 2 Division of Fusion Plasma Physics, KTH Royal Institute of Technology, 100 44 Stockholm, Sweden; 3 Department of Material Science, Metallurgy and Inorganic Chemistry, University of Cádiz, 11003 Cádiz, Spain; 4 Culham Centre for Fusion Energy, Abingdon OX14 3DB, UK; 5 Faculty of Materials Science and Engineering, Warsaw University of Technology, Woloska 141, 02-507 Warsaw, Poland

**Keywords:** JET tokamak with ITER-like wall, dust, ion beam analysis, deuterium, beryllium, tungsten

## Abstract

Silicon plates were installed above the inner and outer divertor of the JET with the ITER-like wall (ILW) after the second and third ILW campaigns to monitor dust generation and deposition with the aim to determine the morphology and content of individual particles and co-deposits, including deuterium content. Particular interest was in metal-based particles: Be, W, steel, Cu. Ex-situ examination after two ILW campaigns was performed by a set of microscopy and ion beam methods including micro-beam nuclear reaction analysis and particle-induced X-ray emission. Different categories of Be-rich particles were found: co-deposits peeled-off from plasma-facing components (PFC), complex multi-element spherical objects, and solid metal splashes and regular spherical droplets. The fuel content on the two latter categories was at the level of 1 × 10^16^ at/cm^−2^ indicating that Be melting and splashing occurred in the very last phase of the second experimental campaign. The splashes adhere firmly to the substrate thus not posing risk of Be dust mobilisation. No tungsten droplets were detected. The only W-containing particles were fragments of tungsten coatings from the divertor tiles.

## 1. Introduction

Plasma-surface interactions (PSI) lead to material erosion, migration of eroded species and their re-deposition thus causing modification of plasma-facing components (PFC) in controlled fusion devices. The intensity of PSI processes may become decisive for the lifetime of wall components in a reactor-class machine. In addition, generation of dust particles associated with erosion-deposition is considered as a potential hazard for reactor safety and economy. It is also a serious issue in the ITER licensing procedure. The JET tokamak with the ITER-like wall (JET-ILW) [1] is a large-scale test bed for plasma operation with walls of the same material configuration as will be implemented in ITER [2]: beryllium (Be) on the main chamber wall and tungsten (W) in the divertor. The JET-ILW research programme has included broad studies of PFC (erosion and fuel inventory) [3,4,5,6,7,8,9,10] and various aspects of dust formation mechanisms, quantities, in-vessel location, morphology, fuel retention, classification (ITER-relevant or only JET-specific), mobilization and generation under water impact [11,12,13,14,15,16,17]. Lessons and previously developed collection methods from other devices have been taken into account [18,19,20,21,22].

In JET-ILW high-resolution photographic in-vessel surveys at shutdowns (inspection of the PFC state including the search for metal splashes) [23,24] are followed by ex situ analyses of particles retrieved by several methods: (i) remotely controlled vacuum cleaning of the divertor tiles, (ii) local sampling with carbon stickers of loosely bound matter from limiter and divertor tiles retrieved from JET, (iii) dust deposited on various erosion-deposition probes located in the divertor and in the main chamber [13,16], and particles mobilized during in-vessel work by the remotely handled (RH) equipment [25]. The amount of dust retrieved from the divertor was at the level of 1 g per experimental campaign comprising 18.9 h (ILW-1 in 2011–2012), 19.5 h (ILW-2, 2013–2014) and 23.3 h (ILW-3: 2015–2016). This is over two orders of magnitude less than removed by vacuum cleaning after the last campaign in JET with carbon walls (JET-C, 200 g) [26].

The composition and internal structure of particles collected on carbon stickers directly from various regions of the JET divertor were studied after the campaigns: ILW-1 [11]; ILW-2 [12,14] and ILW-3 [16]. The search is strongly directed towards objects of particular relevance to ITER: (i) Be- or W-rich flaking co-deposits, (ii) solid metal (Be, W, also Cu) splashes or droplets created in melt events of wall materials and, as a result of damage to components in the neutral beam injection (NBI) systems. Equally needed is the assessment of fuel content in individual grains in order to identify types of particles retaining hydrogen isotopes. Deuterium was studied by ion beam methods in vacuumed [15] and locally sampled dust [14], while tritium in vacuumed matter was determined using radiography [27,28]. All results have coherently indicated that fuel accumulation in the JET-ILW dust was predominantly associated with residual carbon particles being either legacy from JET-C or small debris from the rear side of W-coated carbon fibre composite (CFC) divertor tiles in JET-ILW.

This work is focused on the examination of both individual dust particles and co-deposits collected on silicon (Si) plates of so-called dust monitors located above the JET divertor. Such monitors allow for the characterisation of mobilized particles and co-deposits in the unperturbed form, i.e., remaining in the original state not modified by any external force such as in the case of vacuuming or sampling with sticky pads. It is also stressed that monitors are the only retrievable probes located in the lower part of the main chamber of JET. Therefore, their examination provides unique information on the deposition and fuel retention in that region.

## 2. Materials and Methods

**Figure 1:**
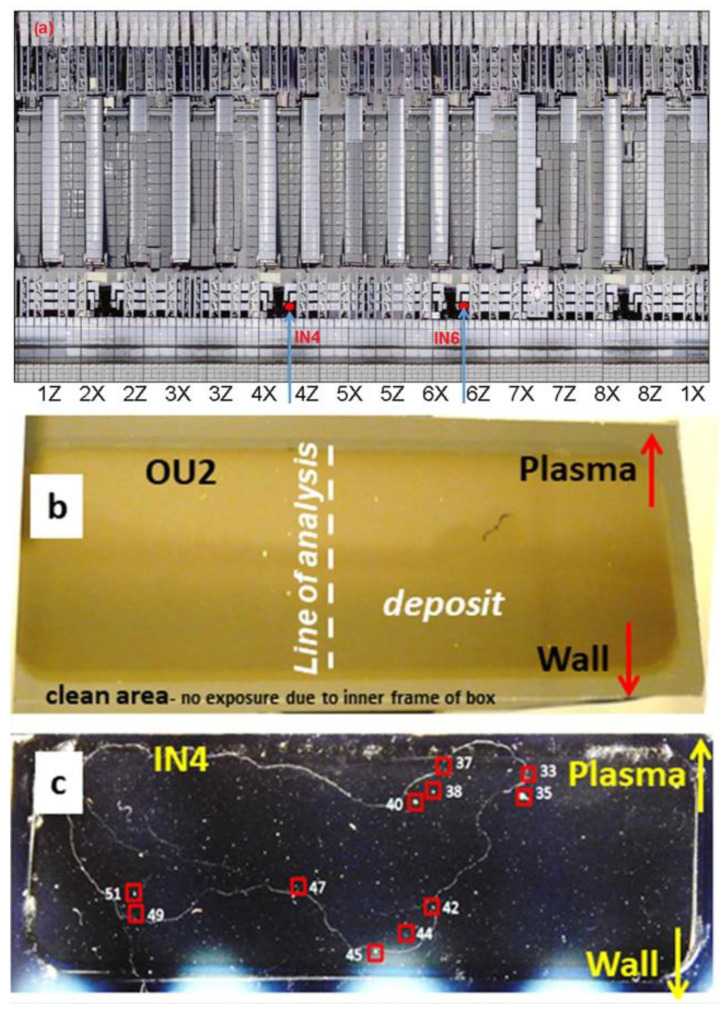
(**a**) Locations of IN4 and IN6 inner Si wafer dust monitors on the inner wall inside the JET vacuum vessel (ILW-2). (**b**) The appearance of the outer wall monitor (OU2) with the marked line of analysis by ToF HIERDA. (**c**) IN4 monitor with marked areas analysed by ion micro-beam, the photographed to enhance surface features. The size of both monitors is 50 × 25 mm. The distance between IN4 and IN6 monitors at (**a**) is about 1.5 m.

The study was carried out on the Si plates of four monitors exposed during ILW-2, i.e., when this type of diagnostic was applied in JET for the first time. In addition, for comparison of fuel retention, two monitors exposed during ILW-3 were examined. They were inserted above the inner and outer divertor tiles but below the arrays of poloidal limiters. Two inner wall monitors (IN4 and IN6) were below the inner wall cladding (IWC), while on the outer wall (OU2 and OU6) they were between the saddle coils below the antenna for ion cyclotron resonance frequency (ICRF) heating. Details about their location and type of analyses are given in Table 1, whereas respective parts of Figure 1a–c show the tile map on the inner wall with marked monitors’ locations and surface features of the OU2 and IN4 plates after retrieval from JET. X and Z on Figure 1a stand for the JET notation of 8 octants of the JET vessel. It is used to indicate the location of the monitors in Octants 4 and 6. By visual inspection very faint deposition patterns could be perceived: smooth surfaces with very few particles, thus indicated small amount and tiny size of the collected grains. The plates in Figure 1b,c were photographed to enhance surface features and reveal the deposition zone on OU2 and the localization of dust grains or their agglomerates on IN4.

The monitors serve as regular wall probes for erosion and deposition studies in JET-ILW. In addition to dust particles sticking to monitors in single events, there are also co-deposits formed steadily during the entire campaign. Therefore, the study was performed by means of electron and ion beam analysis methods in order: (i) to detect individual particles and their areal density, and to determine qualitative and quantitatively their elemental composition including the deuterium content; (ii) to determine the morphology of co-deposits formed on the monitors. Such comprehensive approach has been indispensable to differentiate between the effects of continuously occurring co-deposition and single events of dust sticking to the monitor surfaces.

Microscopy performed on all four dust collectors at the Warsaw University of Technology (Poland) was used to examine the morphology of single dust particles and splashes. Scanning electron microscopy (SEM) using Hitachi SU-8000 FE-SEM (Tokyo, Japan) equipped with energy-dispersive X-ray spectroscopy (EDX) enabling beryllium detection with a Thermo Scientific Ultra Dry (Waltham, MA, USA), type SDD—silicon drift detector. Focused ion beam (FIB, Dual Beam Hitachi NB5000) with a standard EDX system (no Be detection) enabled the study of particles’ internal structure and composition for Z ≥ 4.

Micro-beam ^3^He-based nuclear reaction analysis (^3^He NRA referred to as ^3^He ion microscopy) was performed on IN4 and OU2 at the Ion Microprobe end-station of the Ruder Bošković Institute Tandem Accelerator Facility using a 2.7 MeV ^3^He^2+^ beam 5–6 μm spot size [29]. Particle induced X-ray emission (PIXE) and elastic backscattering spectroscopy (EBS) spectra were measured in 23 areas (11 on IN4 as marked in Figure 1c and 12 on OU2) simultaneously with NRA to determine elemental composition of dust particles, including distribution of deuterium. Details of the experimental setup are given in [14,15].

The composition and depth profiles of species co-deposited on IN6 and OU2 were determined at the 5 MeV Tandem Accelerator Laboratory at the Uppsala University. Time-of-flight heavy ion elastic recoil detection analysis (ToF-HIERDA) with a 12 MeV Si^3+^ beam was used to measure distribution of species (especially low-Z) deposited and co-implanted in the monitors. The probed depth and the depth resolution were 120 nm and 8–10 nm, respectively. NRA with a 2.5 MeV ^3^He^+^ standard beam (0.8 mm) was applied to quantify deuterium and beryllium on monitors exposed during ILW-3. At the same, the spectrum of backscattered ions was used to determine the content of heavy metals.

## 3. Results and Discussion

### 3.1. Morphology of Individual Particles

**Figure 2:**
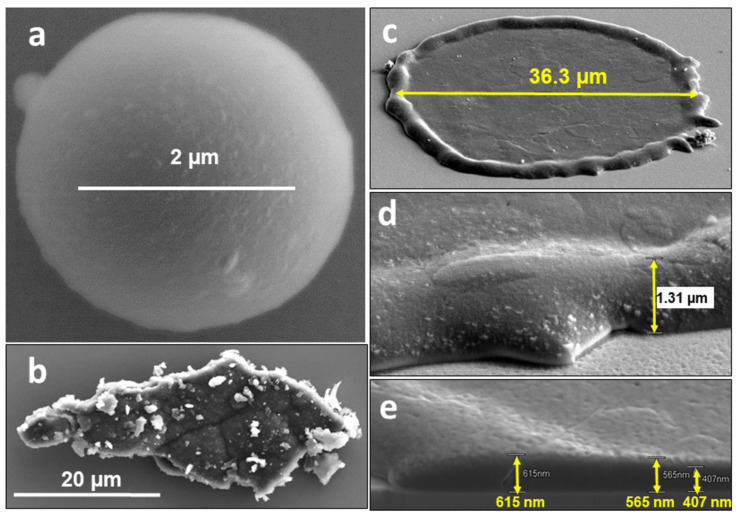
Different categories of Be particles: (**a**) regular spherical droplet, (**b**) co-deposit peeled-off from PFC, (**c**–**e**) circular splash and their geometrical details (IN4 and IN6 ILW-2).

Micrographs in Figure 2a–c show, respectively, different categories of particles on the inner wall monitors (IN4 and IN6): small (2–6 µm in diameter) regular droplets, flakes of co-deposits and splashes. In all of them, Be was identified by EDX as the main component accompanied by oxygen, some carbon content and the presence of Al related to the contamination by the RH operation during the monitors’ retrieval [25]. 

Detailed structure and dimensions of splashes are in Figure 2c–e. The splashes are either circular (“pancake-like” shape surrounded by a 1.2–1.5 µm high rim) with a diameter between 20 µm and 70 µm or of somewhat oval/elongated shape. Their thickness determined from several FIB-produced cross-sections is between 400 nm and 750 nm. Be splashes of larger dimensions (even > 200 µm) were also found on the surface of IN4 collector, as it will be shown later. The surfaces of splashes are free from co-deposits. In addition, geometrical features indicate that molten Be reached the plates at the final stage of discharges when the magnetic field was already weak (elongated splashes) or after the discharge termination (circular objects). Their formation occurred most probably at the last phase of ILW-2 during experiments aiming at the runaway electron generation, which led to disruptions and consequential high heat loads to the upper dump plates (UDP) causing their melt damage and material losses [23]. 

The original volume of the splashed droplets can be assessed in the range 150–2700 µm^3^ (1.5–27 × 10*^−^*^10^ cm^3^), while the small regular droplets are only 4–110 µm^3^. One may tentatively suggest that the latter ones cooled down before reaching the monitor, while the bigger objects hit the Si plates in the molten state.

**Figure 3:**
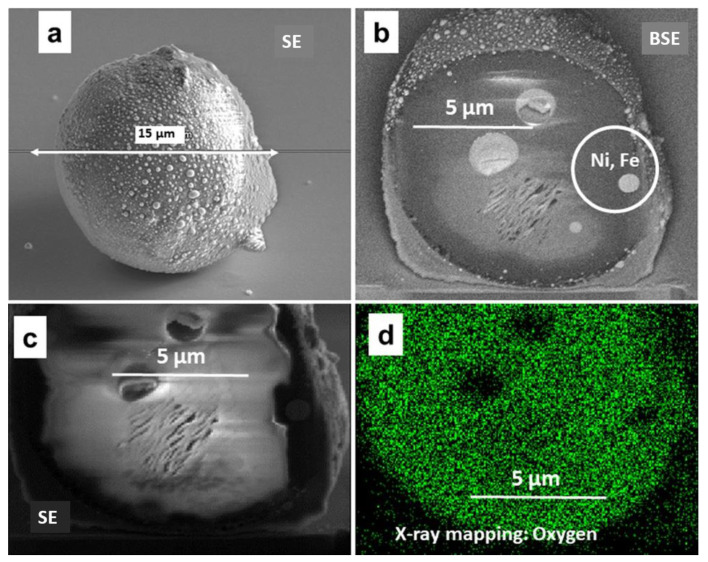
Be-based particle covered by a shell Be-rich co-deposit (IN6 ILW-2). Secondary (**a**,**b**) and backscattered (**c**) electron images, as well as the X-ray map of oxygen on the particle cross-section (**d**). An area enriched with Ni and Fe is marked on (**c**).

Arrival of particles to the Si plates occurred at different stages of ILW-2, not only during the final phase of ILW-2. This statement is supported by the documentation in Figure 3a–c showing secondary (SE) and backscattered electron images (BSE) for the external appearance and FIB-produced cross-sections of a complex particle of spherical shape.

The X-ray mapping on the cross-section, Figure 3d, has found oxygen as the main element with only some traces of Ni and Fe in the entire structure. The result indicates that the oxygen presence is associated with Be, though the EDX system at the FIB apparatus is not capable of detecting that element. A similar situation was reported in [16] where only O could be identified in a particle vacuumed after ILW-1 and studied with a transmission electron microscope. The origin of the complex object in Figure 3 cannot be decided with a satisfactory level of confidence. It may be either a Be droplet or a debris from the limiters. However, it can be stated that the original grain deposited on the monitor remained mobile, and then was fairly uniformly coated by the Be-based deposit with some admixture of Ni and Fe; the composition of deposits is discussed later.

**Figure 4:**
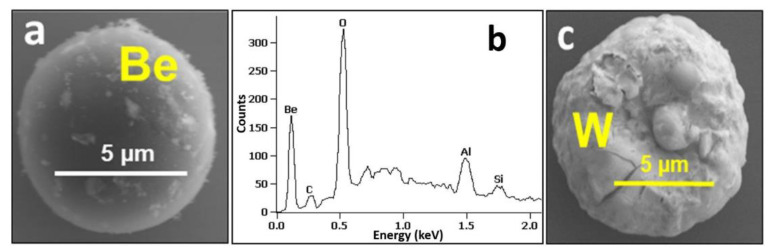
SEM images (**a**,**c**) and EDX spectrum (**b**) of a dust particles present on the outer wall OU6 monitor (ILW-2).

Micrographs and X-ray spectrum in Figure 4 show two examples for particles found on the OU6 surface. EDX has revealed that except Be-based droplets shown in Figure 4a,b and peeled-off co-deposits, several other groups of particles are present on the OU6 and OU2 monitors: molten fragments of W coatings, as shown in Figure 4c; carbon-based particles, aluminosilicates (ceramics), boron nitride from the damaged reciprocating probe, Ni, W and Fe-based. In contrast to the OU2 monitor, a significant number of beryllium splashes was found on the surface of OU6 collector. Most of them were elongated (“pancake-like” shape) with a size between 50 µm and 200 µm. Some of them were cracked thus indicating metal cooling on a cold (around 150 °C) collector. It is stressed that despite internal stresses, the splashes adhere well to the substrate.

**Figure 5:**
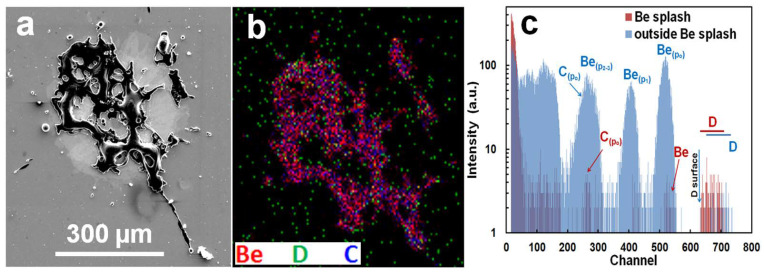
(**a**) Micrograph of large Be splash. (**b**) 2D RGM map from the same particle by ^3^He ion microscopy. (**c**) Related NRA spectra from the Be splash and the surrounding area (IN4 ILW-2).

^3^He ion microscopy was performed on IN4 and OU6 collectors with the aim to identify particles containing larger concentrations of Be, C, and heavier metals such as Cr, Fe, Ni, W and Mo, looking at the same time for the deuterium concentration. Figure 5 shows SEM and IBA data for large Be splash. Minor carbon concentration could be associated with some co-deposits present on Be. Deuterium is detected all over the measured region and it is not associated directly with the Be splash. This is also evident by the comparison of the related NRA spectra (Figure 5c) from the Be splash and the surrounding area round the particle; it is evident that D is present on the Si wafer all over the place.

**Figure 6:**
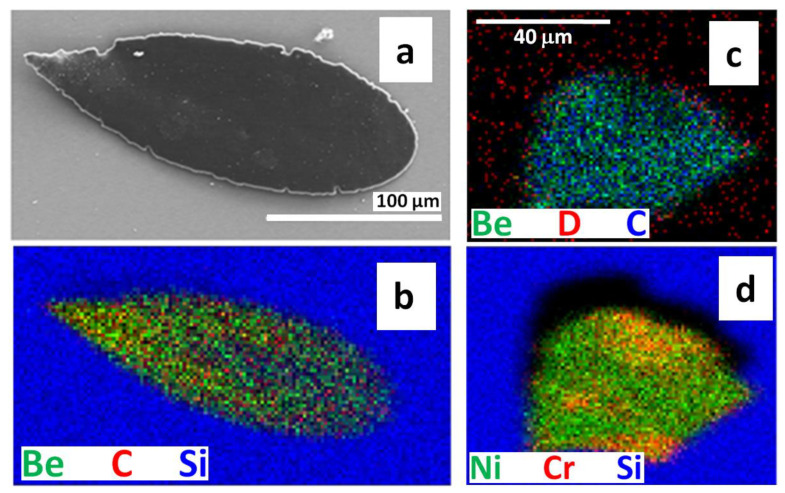
(**a**,**b**) Elongated Be splash, including micrograph and 2D RGM map obtained from the ^3^He ion microscopy. (**c**,**d**) 2D RGB maps of metallic particle. (IN4-ILW-2).

Figure 6 shows an elongated Be splash (a,b) and, in (c,d), an irregularly shaped particle rich in Be and heavier metals. The related 2D RGB maps (Figure 6b,c) show again that D is detected not only on the splashes, but it is spread fairly uniformly over the surface. Quantitative NRA analysis of the Be splash, Figure 6a,b, gives following results: D 0.1 atomic percent (at%), C 2.2 at%, Be 97.7 at% and the thickness of 51 × 10^18^ atoms/cm^2^ corresponding to ~ 4.2 µm assuming Be density of 1.85 g/cm^3^. The estimated thickness of the second particle (Figure 6c,d) is 48 × 10^18^ atoms/cm^2^ with the following composition: D 0 at%, Be 81 at%, C. The remaining 18 at% is associated mostly with oxygen (invisible by NRA but demonstrated by SEM-EDX) and with heavier metals identified by PIXE, with their relative contributions (normalized to 100%) of 3.5% Mo, 75% Ni, 11% Cr, 1.7% Fe, 0.1% Ti, 7% Si and 1.7% Al. We estimate the detection limits of the used NRA setup to ~5 at% for B and C while for D concentrations down to 0.01 at% can be determined. Heavier metals are determined by PIXE and their detection limit is estimated to be between 10 to 100 ppm (parts per million in weight).

The quantitative NRA analysis of the areas surrounding Be particles identified a layer with a thickness of 6.5 × 10^17^ atoms/cm^2^. With PIXE and RBS some minor quantities of Cr, Fe, Ni, W and Mo were detected. These heavier elements could be in the thin deposit or as small particles Inconel dust particles on the Si surface. The relative atomic concentrations (normalized to 100%) of metals could be assessed: Ni 30%, Fe 45%, Cr 20%, Mo 2.4% and W 2.6%.

**Figure 7:**
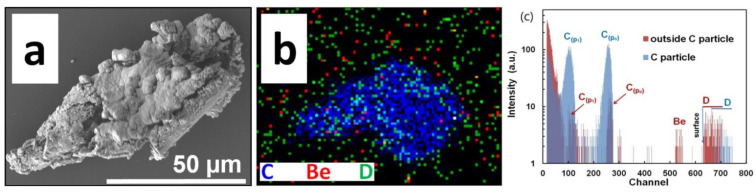
(**a**) Fragment of W coating on CFCs (carbon fibre coating). (**b**) C, Be and D 2D RGM map of carbon rich particle. (**c**) NRA spectra related to the particle and the surrounding area. (IN4).

In addition to metallic objects, a number of C-based particles were found such as the one shown in Figure 7. The detected deuterium, Figure 7b, is not associated with the carbon particle. A comparison of the related NRA spectra (Figure 7c) from the particle and the surrounding area confirms that most of D is on the Si wafer. PIXE spectra from this and all the other analysed regions show the presence of heavier elements (Fe, Ni) spread uniformly over the measured surfaces, suggesting the existence of thin deposits or number of small un-resolvable particles spread round on the Si surface.

### 3.2. Small Particles and Co-Deposits

**Figure 8:**
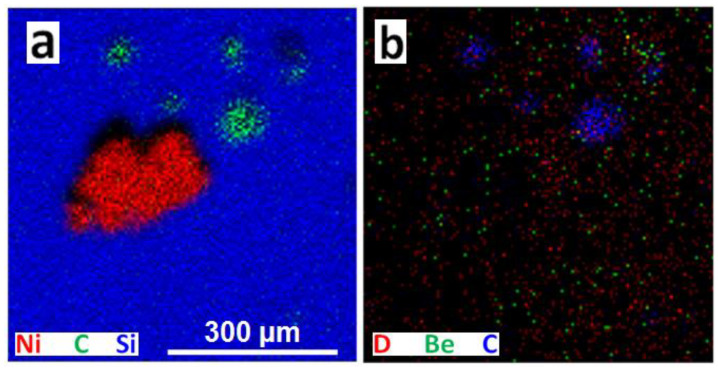
2D RGB maps of an area with a number of particles of various sizes showing one large Ni-Fe-Cr debris or splash (**a**) and several smaller C-rich particles (**a**,**b**). (OU6 ILW-2).

Figure 8 shows an area containing one larger particle with medium-Z metals (Ni, Fe, Cr) and a number of small C-rich particles.

**Figure 9:**
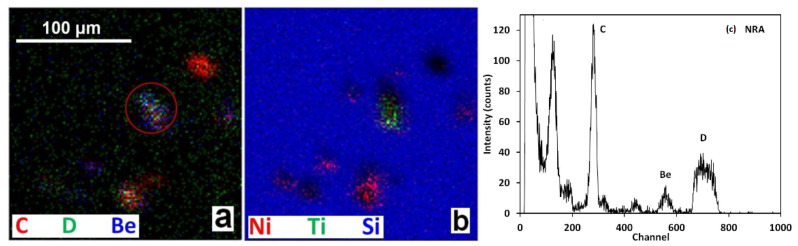
(**a**,**b**) 2D RGB maps of an area with a number of smaller dust particles. (**c**) NRA spectrum of the related area (OU6 ILW-2).

Once more, the presence of D is detected all over the scanned area. Figure 9 shows a number of scattered particles rich either in C or Be, or heavier metals. NRA spectrum in Figure 9c for the whole scanned area clearly shows dominant carbon peak and a presence of deuterium. Quantitative analysis was performed on the encircled particle (Figure 9a). Thickness of that particle is estimated to 12 × 10^18^ atoms/cm^2^ with the following composition: D 2.5 at%, Be 15 at%, C 22 at%, O 8 at%, Si 47.4 at% and the rest is Al, Ti, Cr, Fe, Ni. The reported Si most probably belongs to the Si wafer backing and not to the dust particle itself. Oxygen can also be related to the Si substrate. Its concentration is estimated from the related RBS spectrum. This is the particle that showed the highest D content among all analysed regions at IN4 and OU6 collectors scanned by ^3^He ion microscopy. The analysis of the region at Figure 9 surrounding the visible particles results with identification of a localized layer with thickness of about 6.5 × 10^17^ atoms/cm^2^ and composition: D 6.5 at%, Si 75.7 at%, O 16.2 at% with the rest Cr, Fe, Ni, W. In summary, D contents at the levels between 0.4 to 1 × 10^17^ at/cm^2^ are identified at the surface layers of the IN4 and OU6 monitors at analysed micro-locations surrounding the particles.

**Figure 10:**
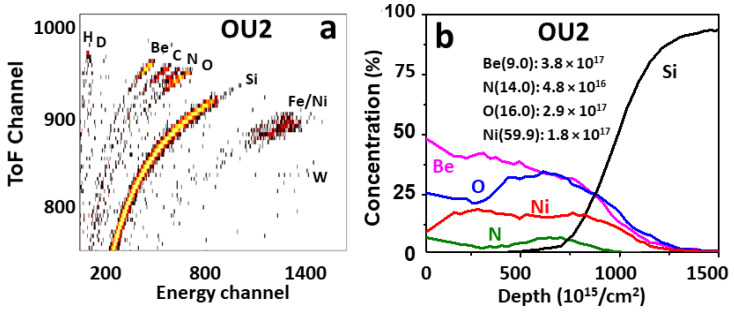
ToF HIERDA spectrum (**a**) and depth profiles of elements (**b**) in co-deposits on the monitor exposed on the outer wall during ILW-2.

**Figure 11:**
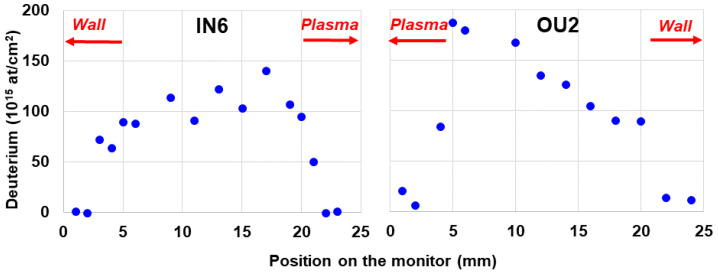
Deuterium distribution along the line of analysis for IN6 and OU2 dust collectors exposed during ILW-3. As indicated, for IN6 zero point corresponds to Wall side, and for OU2 to Plasma side (see Figure 1a,b for clarification).

Standard beam NRA and ToF-HIERDA measurements were performed in several radial positions on plates IN6 and OU2 to study the elemental composition and depth profiles of respective species in co-deposits formed steadily during the entire campaign. HIERDA spectrum and depth profiles of species in co-deposits on the outer wall monitor exposed during ILW-2 are shown in Figure 10a,b. These data are for the position close to the plasma side of the monitors. Measurements in several positions on the outer and the inner monitor have indicated some local differences in the co-deposit thickness, but the qualitative composition on both sides of the divertor has been qualitatively identical: H, D, Be, C, N, O, Fe, Ni and W. Beryllium and oxygen have been the main constituents with the Be:O concentration ratios below 1. Depth profiles of both elements are quite similar and there is no increase of O content at the surface, thus indicating that oxygen was co-deposited and gettered by Be during the tokamak operation. Ni and Fe are the components of Inconel alloy. They most probably originate from the grills of antennas for auxiliary plasma heating. Only traces of W are detected: 1.4 × 10^15^/cm^2^ and 3.8 × 10^15^/cm^2^ on the inner and outer, respectively. The deuterium feature is very weak, while the hydrogen signal is fairly clear due to the fact that the ILW-2 campaign was concluded with approximately 300 shots in hydrogen fuel. This interpretation is supported by the comparison to the situation after ILW-3, when the D contents on both monitors were reaching 2 × 10^17^ at/cm^2^, as shown in Figure 11.

## 4. Conclusions

This work presents the first combined approach to the morphology of both individual dust particles and co-deposits not perturbed by the collection method. The fuel distribution and ITER-relevance of different particle classes have been determined. All categories of Be-based particles presented in the paper are ITER-relevant. Other categories of dust such as debris of CFC, fragments of partly molten W coatings, Inconel splashes and droplets, are of no relevance to ITER, because neither CFC nor W coatings and Inconel will be used in the reactor. The surfaces of Be droplets and splashes are mostly free from co-deposits thus indicating that molten Be reached the monitors at the final phase of the ILW-2 experimental campaign during experiments aiming at the runaway electron generation. As documented by micro-NRA and HIERDA, the deuterium content in dust particles and in co-deposits after ILW-2 is low (up to the levels of ~10^17^/cm^2^); it is related to the fuelling of the last 300 discharges in that campaign with hydrogen. Even after the ILW-3 campaign fuelled entirely with deuterium, the content of that isotope in co-deposits on the monitors did not exceed 2 × 10^17^/cm^2^. In summary, the studies have consistently shown low fuel retention and a very small amount ITER-relevant dust generated in JET-ILW: 5–10% by weight of the total mass (1 g) retrieved from the vessel.

## Figures and Tables

**Table 1:** Analysed Si wafer dust monitors related to this work.

Dust Collector/ILW Campaign	SEM/EDX Microscopy (Surface-Dust)	^3^He Microscopy (Surface-Dust)	ToF HIERDA (Depth Profiles)	^3^He NRA (D Distribution)
Inner 4 (IN4)/ILW-2	X	X		
Inner 6 (IN6)/ILW-2	X		X	X
Outer 2 (OU2)/ILW-2	X		X	X
Outer 6 (OU6)/ILW-2	X	X		
Inner 6 (IN6)/ILW-3				X
Outer 2 (OU2)/ILW-3				X

## Data Availability

The data presented in this study are available on request from the corresponding authors.
